# The data on the removal of turbidity and biological agents in spent filter backwash by bed ceramic in water treatment process

**DOI:** 10.1016/j.dib.2018.06.037

**Published:** 2018-06-21

**Authors:** Reza Sardari, Noushin Osouleddini

**Affiliations:** a Active Pharmaceutical Ingredients Research Center (APIRC), Pharmaceutical Sciences Branch, Islamic Azad University, Tehran, Iran; b Department of Chemistry, Ardabil Branch, Islamic Azad University, Ardabil, Iran

**Keywords:** Ceramic membrane, Wastewater treatment, Biological agents, Spent filter backwash

## Abstract

The use of a ceramic membrane is not only a new and modern technique, but reduce the use of chemicals and coagulants as well, and also having high mechanical and chemical resistance reduces costs over consecutive years. The aim of this research was to remove turbidity and biological agents such as Diatoms, Chlorophyte, Cyanophyceae, Protozoa, and Nematodes by using of ceramic membranes. A ceramic pilot plant was designed and constructed. Titanium oxide (TiO_2_) and aluminum oxide (Al_2_O_3_) ultrafiltration membrane with the length, diameter and pore sizes of 25 cm, 2.7 cm, and 50 nm was used. The inlet flow was the effluent resulted from the backwashing of a sand filter. This data showed that the possibility of removing of this agent was high by comparing the size of the agents and ceramic membrane pore size. Therefore, the construction of a pilot plant of ceramic membranes with 50 nm pore size and dimension (*H* = 1.5 m, *Y* = 20 cm, *X* = 50 cm) was offered a constant flow filtration, and sampling was performed at different times. The results showed that all biological agents except diatoms have a removal efficiency of 100% and the effluent׳s turbidity was 0.1 NTU.

## Specifications Table

**Table:** 

Subject area	Water treatment
More specific subject area	Membrane Technology
Type of data	Tables and Figures
How data was acquired	A ceramic pilot plant was designed and constructed. Titanium oxide (TiO_2_) and aluminum oxide (Al_2_O_3_) ultrafiltration membrane with the length, diameter and pore sizes of 25 cm, 2.7 cm, and 50 nm was used. The inlet flow was the effluent resulted from the backwashing of a sand filter.
Data format	Analyzed
Experimental factors	The biological agents were counted using Sedgewick Rafter cell and microscope.
Experimental features	All of the samplings and analyses were conducted according to Standard Methods for the Examination of Water and Wastewater.
Data source location	Tehran, Tehran province, Iran.
Data accessibility	Data are included in this article

## Value of the data

• Due to the limitations of traditional biological treatment processes such as space requirements, high retention time, and using of chemicals, etc., advanced biological treatment processes such as membrane process have been attracted attention on a global scale.
• This data shows the capability of ceramic titanium oxide (TiO_2_) and aluminum oxide (Al_2_O_3_) ultrafiltration membranes for treatment of turbid water.
• The data in this article showed that the turbidity in the raw wastewater was 230 NTU, that was reduced by the ceramic membrane to an average of 5% of the inlet turbidity.
• The data in this article shows the high efficiency of ceramic membranes in reducing biological agents from turbid water.
• This data indicates the efficiency of ceramic membrane for removing diatoms and chlorophyte with efficiency of 100%.

## 1 Data

The data on the biological agents are presented in Table 1. As can be seen the number of diatoms, chlorophyte, nematodes in the wastewater was 43,400, 350, and 2, respectively. The number of diatoms gradually decreased by passing through the ceramic membrane in a continuous flow. Diatoms that passed through the membrane was in the type of Cyclotella (ring shape), and the removal efficiency of diatoms and chlorophyte was about 100% in the wastewater (Table 2).

**Table 1:** Counting of biological agents in the wastewater after passing through the ceramic membrane.

**Biological organism (unit/1000 ml)**	**The first 10 min**	**The second 10 min**	**The third 10 min**	**The fourth 10 min**	**The fifth 10 min**	**The sixth 10 min**	**Wastewater**
Diatomaceae	32	22	12	5	8	6	43,400
Cholorophyceae	No	No	No	No	No	No	350
Cyanophyceae	No	No	No	No	No	No	No
Protozoa	No	No	No	No	No	No	No
Rotifera	No	No	No	No	No	No	No
Curstaceae	No	No	No	No	No	No	No
Others	No	No	No	No	No	No	No
Nematode	No	No	No	No	No	No	2

**Table 2:** Removal efficiency of biological agents in wastewater using ceramic membrane system.

**Biological organisms (Unit/1000 ml)**	**Removal efficiency (%)**
Diatomaceae	99.7%
Protozoa	–
Nematode	100%
Rotifera	–
Cholorophyceae	100%

**Fig. 1:**
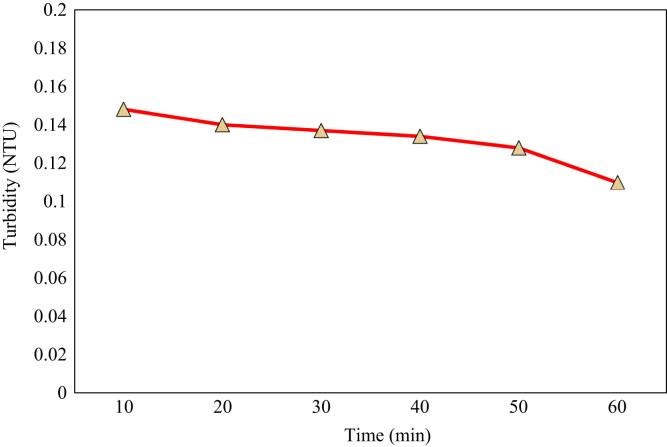
The level of turbidity after passing of stream through the ceramic membrane in the unit of time.

The level of turbidity in the wastewater was equal to 230 NTU. As can be seen by the passing of stream through the ceramic membrane in the unit of time, the level of turbidity is decreased to an average of 5% in which collecting sample is performed every ten minutes (Fig. 1).

**Fig. 2:**
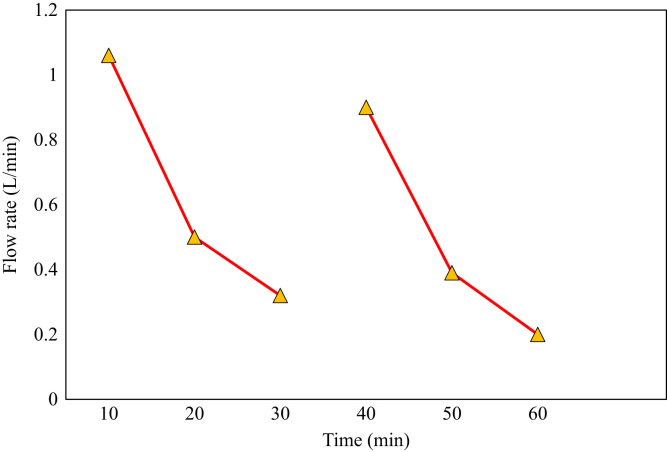
Reducing the amount of output ceramic membranes.

The time of cleaning the membrane was 45 min after continuous treatment; since the output flow rate reaches its minimum value, which reduces the efficiency of the device (Fig. 2).

## 2 Experimental design, materials and methods

In this data, titanium oxide (TiO_2_) and aluminum oxide (Al_2_O_3_) ultrafiltration membrane with the length and diameter of 25, 2.7 cm, respectively, and pore sizes of 50 nm was used. The used sample was the effluent resulted from the backwashing of one of the sand filters of Tehran water Treatment. The chemical was used for the washing of this membrane was sodium hydroxide (NaOH) which will be explained in the following.

All experiments were carried out in a ceramic pilot plant that was designed and constructed by Kahroba Gostar. This system is composed of a pump (type: WB50/037D), a ceramic membrane, pressure gauges, control valves, and a 4-l reservoir.

The procedure had three stages. In the first stage, wastewater treatment was done by the ceramic pilot plant, in the second stage the biological testing and finally, the physical chemistry tests such as the turbidity and pH was done. In the first stage, the wastewater entering the tank and then pump circulate the wastewater within the machine and then pressure is regulated in the 1 bar. The wastewater input was a continuous flow and sampling carried out at certain times (every 10 min). It should be noted that due to the condensed wastewater, the volumetric flow rate at the outlet of the device is reduced and the membrane is washed with sodium hydroxide.

Secondly, collected samples were prepared for biological testing, in a way that 1 l of the sample is passed through 0.8-micron filters. The filter was washed with one mL of distilled water and then entered to the Sedgewick Rafter cell; the counting biological agents was conducted and reported by microscope (MOTIK). The complete result can be achieved by comparing the number of organisms in the wastewater and samples.

In the third stage, Turbidity and pH were measured by a Turbidimeters (N/2100-HACH Company) and a pH meter (Type780-Metrohm Company) in the laboratory.

There are several methods for washing and cleaning of ceramic membrane, reverse wash is one of the important ways in the industry in which treated wastewater entered to the membrane reversely (from outside to inside) again by a membrane, and with the help air compressors, the air pressure was equal to 5 bars. This measure causes a significant part of the blocked pores of the membrane is opened again and reach to initial performance [1], [2], [3], [4], [5], [6].

All of the samplings and analyses were conducted according to standard methods for the examination of water and wastewater [7].
